# What determines prey selection in owls? Roles of prey traits, prey class, environmental variables, and taxonomic specialization

**DOI:** 10.1002/ece3.3899

**Published:** 2018-02-22

**Authors:** Orr Comay, Tamar Dayan

**Affiliations:** ^1^ Department of Zoology and the Steinhardt Museum of Natural History Tel Aviv University Tel Aviv Israel

**Keywords:** body size, Israel, owls, predator specialization, prey selection

## Abstract

Ecological theory suggests that prey size should increase with predator size, but this trend may be masked by other factors affecting prey selection, such as environmental constraints or specific prey preferences of predator species. Owls are an ideal case study for exploring how predator body size affects prey selection in the presence of other factors due to the ease of analyzing their diets from owl pellets and their widespread distributions, allowing interspecific comparisons between variable habitats. Here, we analyze various dimensions of prey resource selection among owls, including prey size, taxonomy (i.e., whether or not particular taxa are favored regardless of their size), and prey traits (movement type, social structure, activity pattern, and diet). We collected pellets of five sympatric owl species (*Athene noctua*,* Tyto alba*,* Asio otus*,* Strix aluco,* and *Bubo bubo*) from 78 sites across the Mediterranean Levant. Prey intake was compared between sites, with various environmental variables and owl species as predictors of abundance. Despite significant environmental impacts on prey intake, some key patterns emerge among owl species studied. Owls select prey by predator body size: Larger owls tend to feed on wider ranges of prey sizes, leading to higher means. In addition, guild members show both specialization and generalism in terms of prey taxa, sometimes in contrast with the expectations of the predator–prey body size hypothesis. Our results suggest that while predator body size is an important factor in prey selection, taxon specialization by predator species also has considerable impact.

## 1 INTRODUCTION

Separation between predatory species in the food niche dimension has been studied extensively in the ecological literature (e.g., Schoener, 1974 and references therein; Kamler, Stenkewitz, Klare, Jacobsen, & Macdonald, 2012; Domingo, Domingo, Badgley, Sanisidro, & Morales, 2013; Symes, Wilson, Woodborne, Shaikh, & Scantlebury, 2013; Sheremetev, Rozenfeld, Dmitriev, Jargalsaikhan, & Enkh‐Amgalan, 2014; Källgren, Pedersen, & Nilssen, 2015), and a general pattern of increased prey size with increasing predator size has been recognized, for example, in numerous guilds of birds, carnivorous mammals, lizards, wasps, flies, beetles, and marine predators (e.g., Hespenheide, 1973 and references therein; Cohen, Pimm, Yodzis, & Saldaña, 1993; Carbone, Mace, Roberts, & Macdonald, 1999; Brose et al., 2006; Costa, 2009; Nakazawa, 2017 and references therein). However, numerous factors affect actual prey intake, including prey availability, the environment, and intensity of competition (e.g., Herrera & Hiraldo, 1976; Kappes, Weimerskirch, Pinaud, & Le Corre, 2011; Levesque, Juniper, & Marcus, 2003; Luiselli, 2006; Tsuruta & Goto, 2007). Hence, any attempt to assess the importance of predator body size in prey selection among guild members must take these potentially confounding factors into account.

Although it is widely recognized that various factors can influence prey selection, in practice it has been difficult to study them because collecting information on prey identity is logistically challenging. An ideal group for addressing this problem is owls (order Strigiformes). Owls form a guild—defined as a group of species exploiting the same class of resources in a similar way (*sensu* Root, 1967; see also Simberloff & Dayan, 1991)—that offer two important advantages for studying the role of predator body size in prey selection: The relative ease of collecting pellets and identifying prey remains and the subsequent plethora of literature on this subject (e.g., Dor, 1947; Glue, 1967; Gotta & Pigozzi, 1997; Hardy, 1977; Hayward & Garton, 1988; Herrera & Hiraldo, 1976; Obuch, 2011, 2014; Romanowski, 1988; Zhao, Song, Liu, & Shao, 2011), allowing comparisons across species, time, and space.

**Figure 1:**
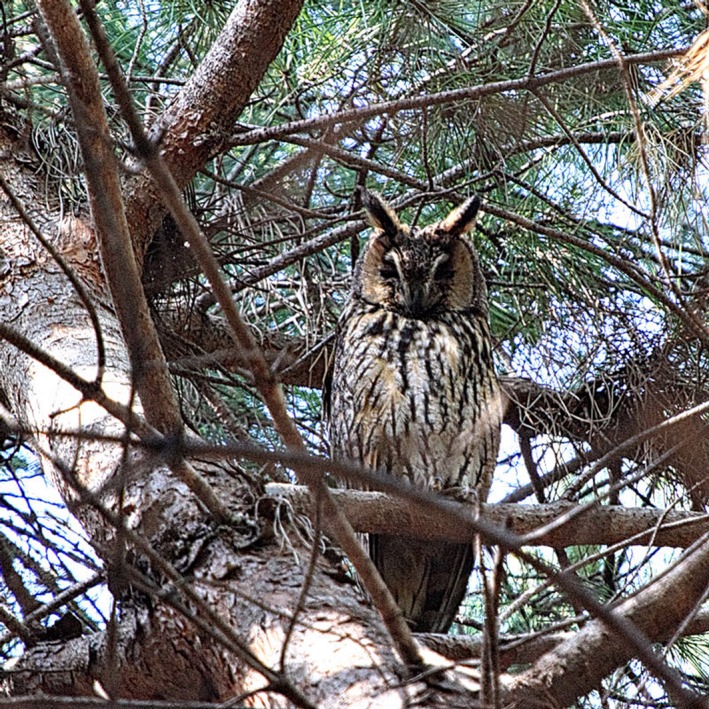
A long‐eared owl (*Asio otus*) from Tel Aviv, Israel. This individual belongs to the country's migrant population of long‐eared owls. Photo credit: Oded Comay

Many studies have addressed prey selection in owl guilds, with conflicting results. While some studies found support for size‐based prey selection among owls, others found contradicting patterns. Yalden (1985) studied *Tyto alba*,* Asio otus* (Figure 1), *Asio flammeus,* and *Strix aluco* in Britain. He found that the largest owl studied (*Strix aluco*) preyed more than the others on relatively large mammals, but also considerably more on invertebrates. In Greece, prey body size differed greatly between the largest (*Bubo bubo*) and the smallest (*Athene noctua*) owls, but not between *Tyto alba* and the larger *Asio otus* (Alivizatos, Goutner, & Zogaris, 2005). That study also found significant intraspecific differences in prey size. A comparison of sympatric *Athene noctua* and *Tyto alba* in Italy found that the larger owl took larger prey items (Gotta & Pigozzi, 1997). Hardy (1977) concluded that while *Tyto alba* hunted smaller prey than the larger *Asio otus*,* Asio flammeus,* and *Strix aluco*, little difference in prey size was found between the latter three species, despite their interspecific size differences. A study in Idaho comparing five species of owls found that the smallest (*Glaucidium gnoma*) and the largest (*Bubo virginianus*) owls differed in prey size from the three intermediate sized species (*Aegolius acadicus*,* Aegolius funereus,* and *Otus kennicotii*)*,* which in turn differed according to habitat but not prey size (Hayward & Garton, 1988). Another study conducted in Idaho found that *Tyto alba* preyed on considerably larger prey than the sympatric and similarly sized *Asio otus*, although the difference they found (42 vs. 31 g) was much greater than in comparable studies elsewhere in North America (Marks & Marti, 1984 and references therein). Thus, while some evidence of the effect of predator size on prey size selection can be discerned, clearly, other selecting factors such as differences in prey taxonomic identity and environmentally driven variation in prey availability must be considered. Consequently, any study of prey selection by owls must be conducted over spatial scales that incorporate such environmental variation.

To address this, here we studied sympatric owl prey diets using prey traits and environmental factors as predictors. We focused on Israel, the West Bank and the Golan Heights, but also examined the generality of our results in light of the ample owl diet literature from around the world. We analyzed the prey of five owl species, spanning an order of magnitude in body mass (from 110 to 1,550 g). Each owl's prey niche is described here in terms of various taxonomic levels (from phylum to species) as well as prey mass and life history traits. We also explored to what extent prey selection is related to environmental variability within our study area and described the emerging patterns of intraguild prey selection independent of the environment (i.e., via a model of prey selection whose predictors are not only owl species, but also environmental variables). Finally, we discuss the implications of our results for understanding the factors driving prey selection in general.

While numerous studies have focused on one or two species (e.g., Balčiauskienė, Jovaišas, Naruševičius, Petraška, & Skuja, 2006; Georgiev, 2005; Gotta & Pigozzi, 1997; Kitowski, 2013; Marks & Marti, 1984; Noland, Maxwell, & Dowler, 2013; Rifai, Al‐Melhim, & Amr, 1998), and a few compared multiple species from multiple sites over a wide area (e.g., Alivizatos et al., 2005; Herrera & Hiraldo, 1976; Obuch, 2011), ours is the first to model the impacts of environmental variables as well as prey life history strategies other than body mass on prey selection.

In this study, we test the hypothesis that prey size (and its amplitude) will increase with predator size within the owl guild, having accounted for various environmental factors affecting prey abundance in the field, prey traits, preferences for particular prey taxa, and other possible sources of bias. To test this idea, we analyze the prey composition of owls across a wide geographical range and incorporate both owl species and the environment as predictors of prey taxonomic composition. A significant effect of the owl species in a model incorporating environmental effects and prey traits on owl prey composition would indicate that owl species prey on different animals not only as the outcome of their environment (and thus, the available prey) but as an inherent trait. Once the existence of different owl prey niches is established, we continue to describe each owl's diet in terms of taxonomic composition and life history strategies of the prey (including mass, but also other traits such as temporal activity patterns, social structure, movement type) in an attempt to rule out the possibility that larger owls favor larger prey for reasons unrelated to their size. For instance, if larger prey and owl species are also more nocturnal than smaller ones, then any apparent predator–prey size pattern could be an artifact of not accounting for temporal activity patterns. In addition to life history traits, we also account for potential taxonomic bias in owl prey selection that may lead to the appearance of predator–prey body size relationships. For example, shrews (Soricidae) are the smallest mammals in our study system, and if smaller owls favor shrews (for reasons other than their size), an artificial predator–prey size relationship could emerge.

## 2 MATERIALS AND METHODS

### 2.1 Study area and sampling design

**Figure 2:**
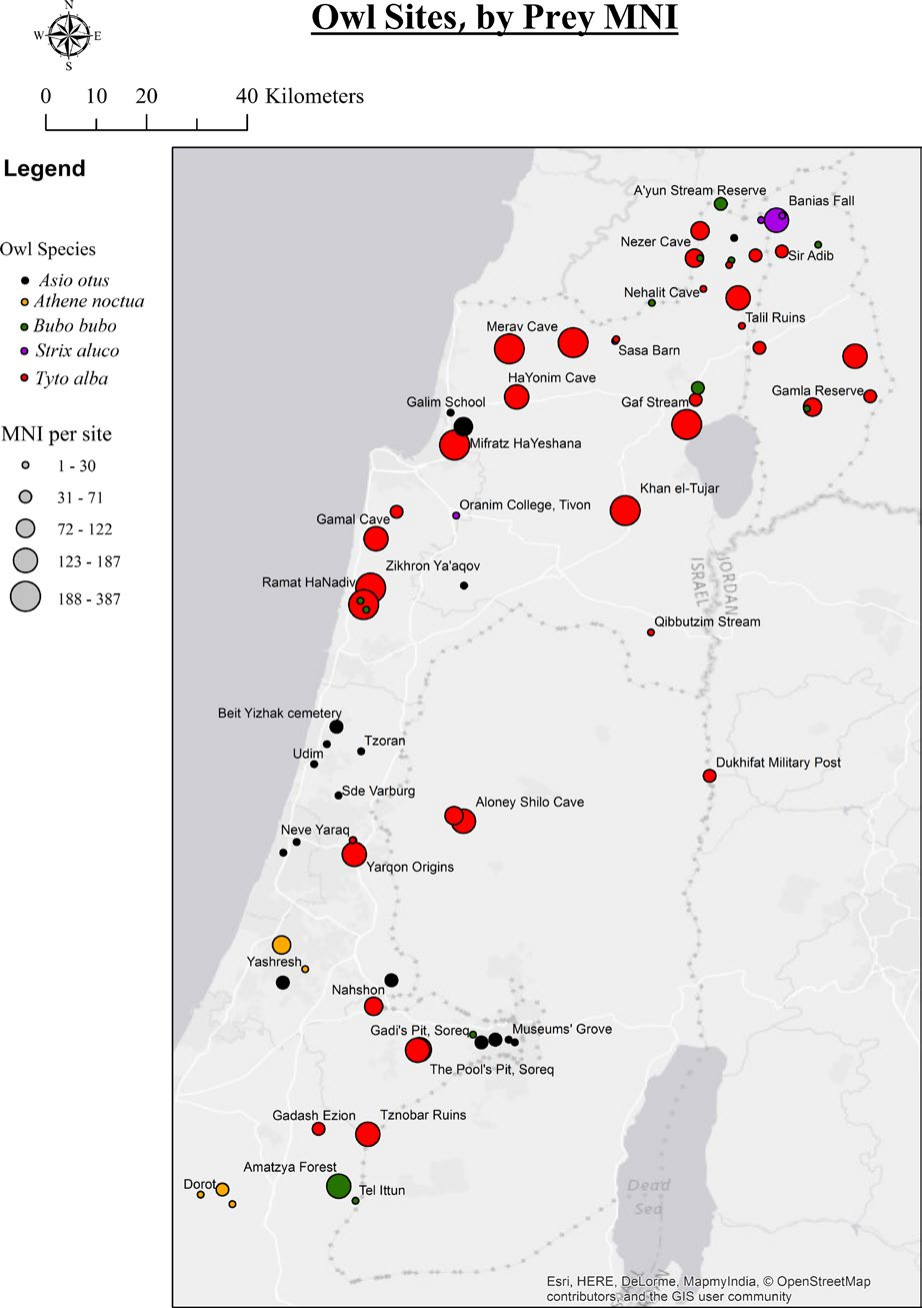
Study area and study sites. Colors indicate owl species, and circle size correlates with prey MNI (minimum number of individuals). See legend

A total of 3,165 owl pellets and bone assemblages were collected from 78 nesting and roosting sites throughout the Mediterranean zone of Israel, the West Bank and Golan Heights (Figure 2; Table 1). The study sites were chosen by their proximity to less disturbed areas, where possible study sites were selected based on detailed knowledge from local naturalists with extensive field experience. They were visited in several field trips in the years 2013–2015 (Israeli Nature and Parks Authority permit numbers 2013/40107, 2014/40572).

**Table 1:** Mammalian and reptilian prey mass (g) per owl species, along with owl body mass data (Paz, 1986). Sample size (*n*) is cranial MNI of all mammalian and reptilian prey individuals that were assigned a mass value (see text). Owl species are ordered by ascending mean prey mass

Owl species	Sites	Owl body mass (g)	Prey MNI with assigned mass	Prey mass (g)			
				Minimum (taxon)	Mean	Median	Maximum (taxon)
*Athene noctua*	5	110–135	167	2 (*Suncus etruscus*)	22.4	14	150 (*Rattus rattus*)
*Tyto alba*	36	250–310	4,370	2 (*Suncus etruscus*)	33.4	28	250 (*Rattus* sp.)
*Asio otus*	18	200–390	429	2 (*Suncus etruscus*)	58.2	14	585 (*Erinaceus concolor*)
*Strix aluco*	6	430–520	66	2 (*Suncus etruscus*)	112.8	110	250 (*Rattus* sp.)
*Bubo bubo*	13	950–1,550	283	2 (*Suncus etruscus*)	143.6	70	585 (*Erinaceus concolor*)

Additionally, the contents of 475 owl pellets and prey assemblages from the Steinhardt Museum of Natural History at Tel Aviv University were added to the database. These pellets (collected in the 1980s–2000s) were significant to the analysis, as they greatly expanded the sample size of the largest owl studied, the Eurasian eagle owl *Bubo bubo*.

### 2.2 Taxonomic identifications and species counts of vertebrate prey

Skulls, mandibles (regardless of preservation state), and hedgehog (Family: Erinaceidae) skins were used for morphological taxonomic identification (Jenrich, Löhr, & Müller, 2012; Niethammer & Krapp, 1978; Qumsiyeh, 1996; Shalmon, Kofyan, & Hadad, 1993; Yorulmaz & Albayrak, 2009) to the lowest possible taxonomic level. When morphological evidence only allowed identification to the genus level, species could still often be identified based on their known geographical distributions (e.g., sites where only one of two closely related species is known to occur (Mendelssohn & Yom‐Tov, 1988; Shalmon et al., 1993; Figure 2)).

### 2.3 Prey mass

We used mass (g) as our measure of prey body size. Adult body mass of all 38 mammalian and reptilian prey species and genera identified as prey items was retrieved from the Israeli literature (Arbel, 1984; Mendelssohn & Yom‐Tov, 1988; Shalmon et al., 1993), as prey size may vary between regions and we needed local measurements. As *Strix aluco* preyed on more birds than other owls (Figure 4), and many of those were not identified to the genus level or lower, relatively few *Strix aluco* prey specimens were assigned mass. When the prey taxon could only be identified to the genus level, we used the mean of all the species belonging to this genus that occur in the study area. This averaging was conducted for specimens of *Pipisterllus* (two specimens, five local species), *Crocidura* (all 483 specimens, four local species), *Rhinolophus* (one specimen, six local species), *Mus* (all 1,814 specimens, two local species), *Gerbillus* (16 specimens, six local species), *Apodemus* (76 specimens, two local species), *Meriones* (11 specimens, three local species), and *Rattus* (126 specimens, two local species), listed here from the lightest genus to the heaviest. A total of 316 specimens were attributed to taxonomic groups higher than the genus level and were excluded from the prey mass analysis.

### 2.4 Invertebrate prey

Invertebrate prey individuals were identified to their class (mainly arthropods, but also three gastropods), but were not identified to lower taxonomic levels, nor were they counted, because the preservation state of their remains rendered the identification and counting process extremely tentative and difficult. Nevertheless, as invertebrates were the smallest prey taken, we wanted to test whether smaller owls prey more on invertebrates than larger ones, and hence their quantification was required. Thus, instead of using the minimum number of individuals (MNI; calculated as the greatest number of paired skeletal elements of a given species divided by two), we simply calculated the proportion of pellets containing arthropod remains. For the purpose of this specific analysis, only whole pellets were examined and bone assemblages were excluded.

### 2.5 Vertebrate prey traits

In order to assess the contribution of prey size in isolation from unrelated traits affecting prey selection, we included prey traits in the owl prey model. For instance, if some owls specialize on volant prey (bats, birds) and these taxa have smaller mass for a given size, then failing to account for volancy could lead to spurious association between predator and prey size. We used the following prey species traits as predictors of abundance in owl diets in interaction with the other predictors stated above: mass (g), diet (granivore, insectivore, grazer), movement type (fly, jump, climb, and burrow), social structure (solitary, social), and diel activity pattern (diurnal, nocturnal). All traits were inferred from the species descriptions in Israel (Mendelssohn & Yom‐Tov, 1988; Paz, 1986; Shalmon et al., 1993). Table 2 details the traits assigned to each of the main prey taxa of owls in this study.

**Table 2:** Common prey taxa traits that were used as predictors for abundance. Mass in grams. See text

Taxon	Mass	Diet	Movement	Sociality	Activity
*Acomys dimidiatus*	40	Granivore, insectivore, grazer	Jump, climb	Social	Nocturnal
*Apodemus flavicollis*	20	Granivore, insectivore, grazer	Jump, climb, burrow	Social	Nocturnal
*Apodemus mystacinus*	35	Granivore, insectivore	Jump, climb, burrow	Social	Nocturnal
*Crocidura*	7	Insectivore	Burrow	Solitary	Nocturnal
*Erinaceus concolor*	585	Insectivore, grazer	Climb, Burrow	Solitary	Nocturnal
*Meriones tristrami*	70	Granivore, grazer	Burrow	Solitary	Nocturnal
*Microtus guentheri*	44	Granivore, grazer	Burrow	Social	Nocturnal, diurnal
*Mus*	14	Granivore, insectivore, grazer	Burrow	Social	Nocturnal, diurnal
*Passer domesticus*	27	Granivore, insectivore, grazer	Fly	Social	Diurnal
*Rattus*	150	Granivore, insectivore, grazer	Jump, climb	Social	Nocturnal
*Spalax ehrenbergi*	150	Granivore, grazer	Burrow	Solitary	Diurnal
*Suncus etruscus*	2	Insectivore	Burrow	Solitary	Nocturnal

### 2.6 Environmental analysis

To account for the potential effects of the environment on prey availability, we quantified the environmental characteristics of each sampling site using the following protocol.

#### 2.6.1 Vicinity

We extracted environmental data (see below) from a four km radius area around each nesting and roosting site (referred to here as its “vicinity”), using ArcGIS 10.4 (ESRI 2015). This radius was chosen as a rule of thumb, as the hunting ranges of owls in Israel were studied both with radio telemetry and GPS transmitters, with the latter suggesting larger hunting ranges than the former (Charter, 2016; Motro, 2011). While telemetric studies suggested a hunting radius of ~500 m for *Tyto alba* (Motro, 2011), recent data from GPS‐tags indicate ranges of 6–8 km and up to 14 km for the same species. Given these results, the range of four km was used a compromise, not only for *Tyto alba* but for all owl species (for which no equivalent data were available). Spatial data were transformed to raster grids with cell size of 100 × 100 m, as detailed below. Numerical values were attributed to cells, and each owl site was attributed a Vegetation Index (see below) calculated based on all cells in its vicinity, regardless of their relative distance.

#### 2.6.2 Geographical analysis

The following layers were downloaded from Open Spaces Portal (Israel Ministry of Environmental Protection 2016): Land uses in Israel (HaMaarag 2016) and Mediterranean vegetation mapping (Nature and Parks Authority 2016). The former includes the following land uses forest/maquis, shrubland, grassland, desert open space, field crops, plantations, built‐up/water, and other open spaces. Water and built‐up areas were differentiated using the World Water Bodies layer (DeLorme Publishing Company 2016). The Mediterranean vegetation mapping layer includes more fine‐scale information of habitats, including park–forest/maquis and low‐density shrubland, high‐density forest and maquis, high‐density maquis, medium‐density maquis/medium–high‐density shrubland, grassland/sparse shrubland and semi‐arid grassland/sparse vegetation/exposed soil. These separate polygonal layers were converted to raster layers with 100‐m edge length cell size and combined to create a layer of land cover, from which we calculated the Vegetation Index (see below).

#### 2.6.3 Vegetation Index

For the purpose of this analysis, we devised a Vegetation Index to represent the key stages of botanical succession in the Mediterranean region of Israel: dwarf‐shrub steppe, garrigue, maquis, or forest, from the sparsest to the thickest, in successional order (Waisel, 1991). Each undeveloped land grid cell in the study area was assigned a value of 1–3, based on these succession stages. Built cells were assigned a value of zero. Cultivated fields were assigned a value of 1. Water cells (sea or fresh waters) and cells without data (outside the spatial extent of the layers, e.g., in Lebanon) were excluded. Afterward, we calculated the Vegetation Index as a weighted average of all the cells’ values in a site's vicinity by the following formula, using the total number of cells in each category:

$$\begin{array}{cc} & \text{Vegetation Index}\\ & \;=\frac{\left(\text{dwarf shrub steppe}\right)+2\cdot \left(\text{garrigue}\right)+3\cdot \left(\text{maquis or forest}\right)}{\text{Total no. of land cells}}. \end{array}$$

The Vegetation Index itself is a dimensionless quantity. It ranges from 0 to 3 for every possible site, with 0 meaning all land cells in its vicinity are built up and 3 meaning they are all covered in maquis or forest.

#### 2.6.4 Climate

Climatic variables were downloaded from worldclime.org (Hijmans, Cameron, Parra, Jones, & Jarvis, 2005). The following variables were attributed to each site: mean annual precipitation (mm), mean annual temperature (°C; referred hereafter as “temperature”), mean temperature of the coldest quarter (°C; referred to hereafter as “winter temperature”), and mean temperature of the warmest quarter (°C; referred to hereafter as “summer temperature”).

### 2.7 Statistical analysis

We used PAST version 3.12 (Hammer, Harper, & Ryan, 2001) and the R language (R Core Team 2016) for conducting statistical tests.

The procedure detailed below follows the recommendations for model‐based thinking in community ecology (Warton, Foster, De'ath, Stoklosa, & Dunstan, 2015) and the mvabund package documentation. We used the mvabund (Wang, Naumann, Wright, & Warton, 2012, 2017) package for R to create a model of prey abundance in owl diets by owl species and the environmental variables detailed above. MNI counts of prey taxa occurring more than four times were used as the response variables, assuming a negative binomial distribution for model errors. This assumption was verified via visual inspection of the Dunn–Smyth residuals *vs*. the linear predictor value plot. The unequal sampling effort (i.e., unequal prey MNI) between sites was taken into account by adding the total MNI of all prey species at each site as an offset term to the model (as recommended by Warton, Foster et al., 2015). This resulted in modeling the effects on relative rather than absolute abundances. We tested the statistical significance of the predictors and removed nonsignificant predictors from further analysis, starting with the full model and using a stepwise backward deletion of predictors, until only significant (α = 0.05) predictors remained. Next, we used the LASSO algorithm (Osborne, Presnell, & Turlach, 2000) to penalize the coefficients of the significant predictors found in the stepwise backward deletion, as recommended by the mvabund package documentation. In sum, the LASSO algorithm adjusts predictors’ coefficients in accordance with their correlation to the response variable (in our case, each species’ abundance in owl diets). Predictors with little correlation to the response could be reduced to zero, thus removing them from the model altogether.

#### 2.7.1 Prey traits as predictors of relative abundance in owl diets

“Fourth corner” models in community ecology are models that examine the effects of species traits on their abundance (Warton, Foster et al., 2015; Warton, Blanchet et al., 2015). While standard community ecology models study predictor‐by‐species effects (e.g., how are micromammal species affected by vegetation density), “fourth corner” models focus on predictor‐by‐trait effects (e.g., how does species size affect its abundance along a temperature gradient). Categorical traits were converted to binary (true/false) variables for the purpose of this analysis, which allowed attributing several (for instance) movement types to each taxon, when appropriate. We used the mvabund R package (Wang et al., 2012, 2017) for analysis. The fourth corner coefficients were plotted using the lattice R package (Deepayan, 2015).

Taxonomy‐based prey selection was analyzed by chi‐squared tests using adjusted residuals for post hoc analysis (Sharpe, 2015).

## 3 RESULTS

A full list of the vertebrate prey taxa (by MNI) found in each site is available at Dryad.org, https://doi.org/10.5061/dryad.9m84np6.

### 3.1 Predictors of owl prey

**Figure 3:**
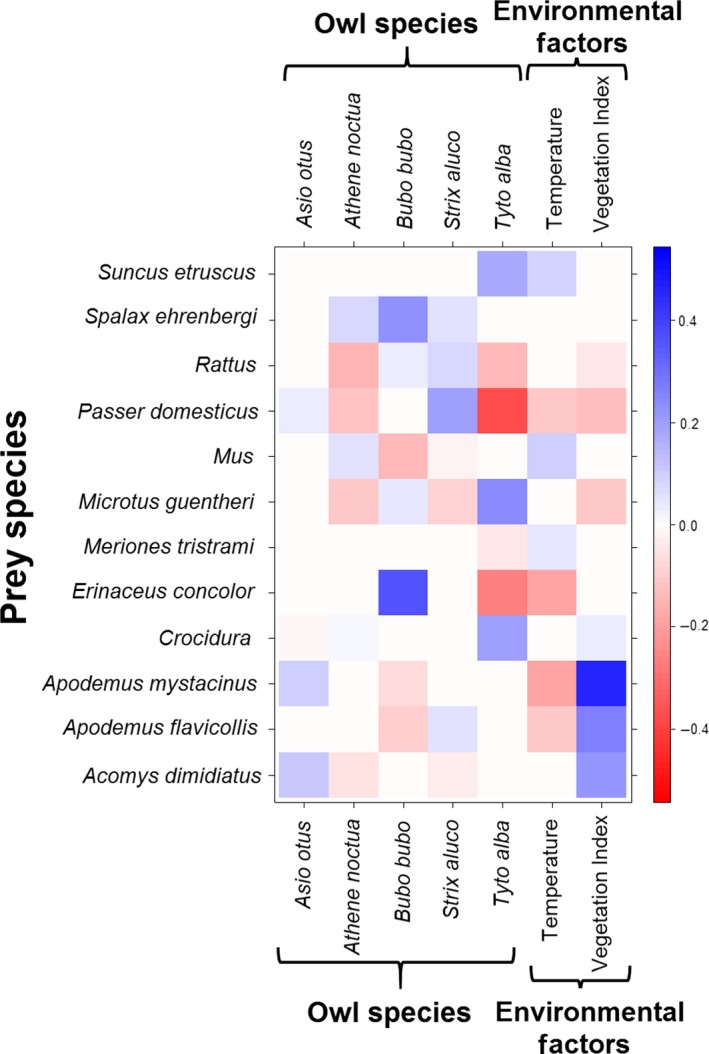
LASSO‐penalized coefficients of prey taxa vs. owl species and the significant environmental predictors (fourth corner model). Colors indicate the effect size from negative (red) to positive (blue) coefficients. Absolute values of coefficients are not comparable between predictors due to differences in units. See text

**Figure 4:**
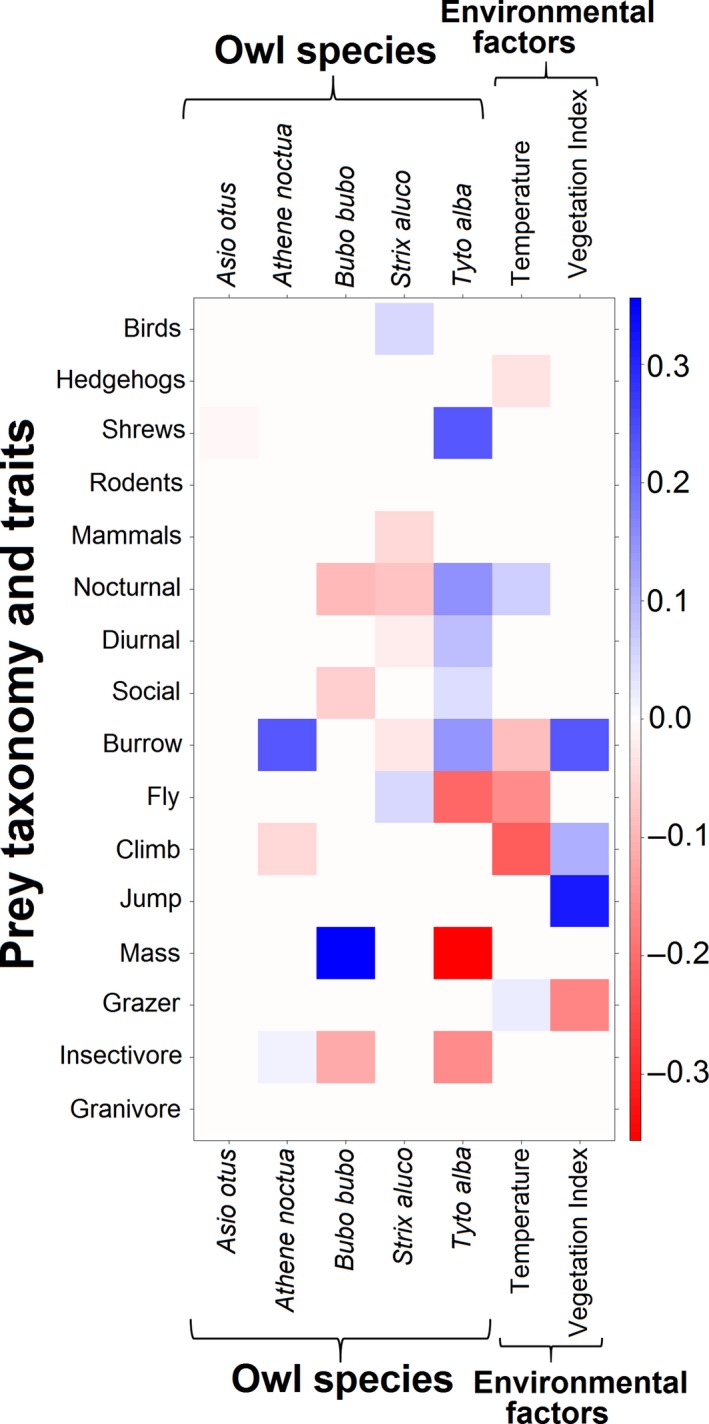
LASSO‐penalized coefficients of prey traits and taxonomy vs. owl species and the significant environmental predictors (fourth corner model). All traits but mass (g) are binary. Colors indicate the effect size from negative (red) to positive (blue) coefficients. Absolute values of coefficients are not comparable between predictors due to differences in units. See text

Owl species, temperature, and the Vegetation Index significantly contributed to prey selection of owls, indicating both an environmental impact and an inherent prey selection pattern among owls, independent of external factors (Table 3). The LASSO‐penalized coefficients of each significant predictor on prey abundance are depicted in Figure 3 (by prey taxon) and in Figure 4 (by prey traits and taxonomy—a “fourth corner” model). Prey mass differed considerably between owl species but not with environmental gradients (Figure 4). Some prey taxa (*Apodemus* spp. and *Acomys dimidiatus*) varied strongly in their abundance between owls of different habitats, but only marginally between owl species; most prey species, however, demonstrated the opposite pattern (Figure 3).

**Table 3:** *p*‐values of the predictor variables tested for statistical significance in owl prey taxa abundance

Variable	Full model *p*‐value	2nd model *p*‐value
Intercept	.52647	.046953
*Athene noctua*	.00200	.001998
*Bubo bubo*	.00699	.014985
*Strix aluco*	.02298	.017982
*Tyto alba*	.00300	.000999
Longitude	.29071	Excluded
Latitude	.06494	Excluded
Mean annual precipitation	.20879	Excluded
Mean annual temperature	.01998	.042957
Winter temperature	.07093	Excluded
Summer temperature	.05594	Excluded
Vegetation Index	.03397	.004995
Total model *p*‐value	.002	.001

### 3.2 Vertebrate prey mass

Prey mass differed between all owl species except between *Strix aluco* and *Bubo bubo* (Table 4). Prey mass mean and standard deviation increased with owl mass on the log_10_ scale (for prey mass mean: Pearson *r*:* R* ^2^ = .92619, *p*‐value = .023805; for prey mass standard deviation: Pearson *r*:* R* ^2^ = .89772, *p*‐value = .03866).

### 3.3 Arthropod prey

**Figure 5:**
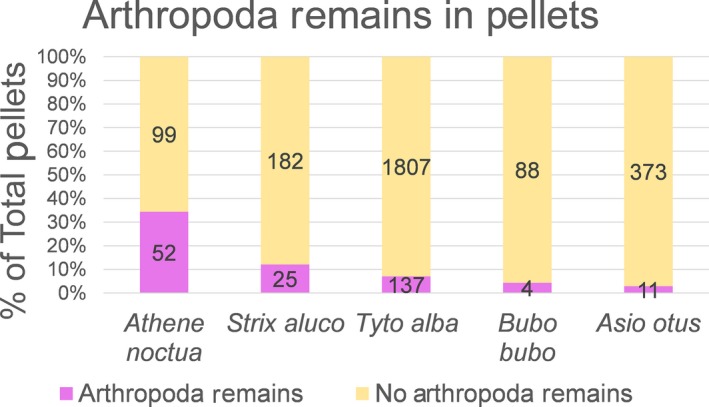
Presence/absence of arthropod remains in owl pellets (binary per pellet). Owl species are ordered by descending arthropod remains content

Figure 5 depicts the proportion of pellets containing arthropod remains of the total analyzed pellets of each owl species. All owl species preyed on arthropods, to significantly varying degrees (χ^2^ test, *p* < 10^−4^). Cells whose adjusted residuals were greater than 3 in absolute value were considered as significantly different from the null hypothesis of equal arthropod consumption (Sharpe, 2015). *Athene noctua* was the most insectivorous owl species examined, with 34.4% (52 of 151) of its pellets containing arthropods remains (adjusted residual = 12.04). *Asio otus* was the least insectivorous owl, with only 2.9% (11 of 384) of its pellets containing arthropod remains (adjusted residual = −4.13). Lack of arthropod remains was also significant in *Tyto alba* pellets (adjusted residual = −3.50). Results for *Strix aluco* (adjusted residual = 2.08) and *Bubo bubo* (adjusted residual = −1.38) were nonsignificant.

**Table 4:** Mann–Whitney *U* test significance levels (*p*‐values) for median vertebrate prey mass differences between owl species, after Bonferroni correction for multiple tests. Significant results are colored in red

	*Asio otus*	*Athene noctua*	*Bubo bubo*	*Strix aluco*
*Athene noctua*	2.56 · 10^−12^			
*Bubo bubo*	3.30 · 10^−15^	1.58 · 10^−30^		
*Strix aluco*	2.55 · 10^−4^	4.00 · 10^−17^	1	
*Tyto alba*	3.84 · 10^−6^	1.48 · 10^−6^	1.89 · 10^−51^	1.93 · 10^−8^

## 4 DISCUSSION

We found a strong relationship between predator body size and prey size and amplitude. These results suggest a significant pattern of prey selection between sympatric owl guild members, despite considerable dietary overlap and environmental effects on prey availability. Prey mass had the greatest effect on predation by owls, more than any other prey trait or environmental factor (Figure 4): prey size mean and variance increased with predator size. Additionally, owls specialized on specific taxa to a varying degree. Some taxon specializations contradicted expectations of the predator–prey size hypothesis: *Tyto alba* (rather than the smaller *Athene noctua*) specialized on the smallest vertebrate prey (shrews, Soricidae; Figure 4) and arthropod consumption did not correlate well with owl size (Figure 5).

Owl body size plays a major role in prey selection, as prey mass had large coefficients in our prey‐by‐trait fourth corner model (Figure 4). Both the mean and the variance of prey mass increased with predator size (Table 1), as expected by theory (Cohen et al., 1993). Notably, all owl species, regardless of size, capture small prey items (arthropods and vertebrates up to 28 g; Table 1, Figure 5). That is, large owls do not simply give up small prey items in favor of large ones, but their size allows them to take large prey items which are apparently unobtainable for smaller owls, in addition to the smaller ones (thus the difference in prey mass variance).

These results accord with those of previous works. For instance, Hardy (1977) found that *Strix aluco* (the largest owl he studied) preyed more often on large animals than *Asio otus* and *Tyto alba*. Capizzi and Luiselli (1998) found that among sympatric *Tyto alba*,* Asio otus,* and *Strix aluco*, the larger owl species took significantly heavier prey than the smaller ones. Alivizatos et al. (2005) compared diets of *Athene noctua*,* Tyto alba*,* Asio otus,* and *Bubo bubo* in Greece. Their results indicate the same pattern: While all owl species captured small prey items (few grams), medium‐sized (100–200 g) animals were not taken by *Athene noctua* and large ones (more than 200 g) were only taken by *Bubo bubo*. In a study of eight owl species in Slovakia, Obuch (2011) similarly found that the largest owl, *Bubo bubo*, feeds on larger prey items than other owls.

However, other works also found contradicting results. Leader (2003) found that *Tyto alba* in the Negev desert feed on larger prey than the sympatric (larger) *Asio otus*. Another interesting point is the two smallest vertebrate prey categories (0–8 g), composed of shrews and bats: *Tyto alba* preys on these taxa more than the smaller owl, *Athene noctua* (Figure 4). Thus, we suggest that other factors besides prey size effect prey selection, as discussed below.

While predator body size is significant for understanding prey selection among owls, it cannot account for taxon‐specific preferences. While *Athene noctua*'s preference for arthropods (Figure 5) may be explained by its small size, *Strix aluco*'s greater intake of avian prey (Figure 4) cannot. More strikingly, shrews (Soricidae), the smallest vertebrate prey, are not favored be *Athene noctua* but by the larger *Tyto alba* (Figure 4).

Past works showed similar patterns to those reported here; that is predator size is important in owl prey selection yet this trend is limited by taxon specializations. Several studies report *Strix aluco* preying on more invertebrates than smaller sympatric owls (Hardy, 1977; Herrera & Hiraldo, 1976; Obuch, 2011; Siracusa, Sarà, La Mantia, & Cairone, 1996; Yalden, 1985). The relatively large intake of birds by *Asio otus* and *Strix aluco* was also reported elsewhere (Hardy, 1977 and references therein; Yalden, 1985; Bertolino, Chiberti, & Perrone, 2001; Kiat et al., 2008; Birrer, 2009; but see Davorin, 2009 and Obuch, 2011). *Tyto alba*'s preference of shrew prey was also found in previous studies comparing sympatric owls (Capizzi & Luiselli, 1998; Georgiev, 2005; Kitowski, 2013). Therefore, our results— the overbearing importance of body size and the taxonomic specializations that contrast its general trend—likely represent a general pattern in owls (and potentially in other guilds as well) and should not be regarded as unique to our study area.

Although the positive association between predator size and prey size has been extensively demonstrated and reviewed in varied ecosystems (Brose et al., 2006; Cohen et al., 1993; Naisbit, Kehrli, Rohr, & Bersier, 2011; Nakazawa, 2017; Schoener, Roughgarden, & Fenchel, 1986) and even in the fossil record (Klompmaker, Kowalewski, Huntley, & Finnegan, 2017), to the best of our knowledge, this study is the first to point out and discuss deviations from this general pattern within a guild of closely related predators. Previous authors emphasized the importance of prey handling mechanism and apparatus (Hespenheide, 1973; Nakazawa, 2017 and references therein), phylogeny (Naisbit et al., 2011), ecosystem type (marine, aquatic, terrestrial etc.; Brose et al., 2006), predator and prey physiology (e.g., endothermy vs. ectothermy; Cohen et al., 1993), and prey availability (Nakazawa, 2017) as factors shaping predator–prey size ratios. None of these factors can explain the unexpected deviations from the general predator–prey size pattern in our study, as the predators we studied are members of the same guild (i.e., they all hunt nocturnal, terrestrial prey by a sudden strike with their talons from the air) and the same order (Strigiformes), all have similar physiologies (endothermic, volant vertebrates) and, to the extent that the environmental variables included our models account for variation in prey availability, differences in prey abundance also seem unlikely to explain our results. Moreover, despite the importance of individual variation in body size (rather than averaged interspecific size differences; Nakazawa, 2017), it could not explain why certain owl species preferred prey taxa in contrast to the general predator–prey size hypothesis. In other words, the specializations (Figures 4 and 5) of *Tyto alba* on shrews and that of *Strix aluco* on non‐mammalian prey (arthropods, birds) cannot be explained by any of the factors suggested in the ample predator–prey size literature.

The inability to explain deviations from the predator–prey size hypothesis by broad ecological mechanisms possibly reflects long‐lasting phylogenetic trajectories that are not easily overshadowed by size trends, even within a guild of closely related predators. In our case, the fact that *Tyto alba* (Tytonidae) takes more shrews (small prey) than the sympatric, smaller *Athene noctua* (Strigidae) and also more jirds (*Meriones* spp.; large prey) than sympatric, larger *Asio otus* (Strigidae; Leader, Yom‐Tov, & Motro, 2010) may reflect a genuswide preference for hunting small, terrestrial mammals (compared with Strigidae) and not the impacts of body size. For instance, a study in Cameroon found that the smaller *Tyto capensis* hunted more shrews but fewer insects than the larger, sympatric *Bubo africanus* (Riegert, Sedláček, & Hutterer, 2008). A study on sympatric *Tyto tenebricosa* and *Ninox strenua* in Australia found that the Tytonid took more mammals, but fewer birds and invertebrates, than the larger Strigid (Bilney, Cooke, & White, 2011). Similarly, the generalistic and adaptable diet displayed by *Strix aluco* compared to that of other sympatric owls (Figure 4; Obuch, 2011) is more likely a common trait of the genus than a consequence of this species’ relatively large size in our case study. This is confirmed by the relatively low numbers of mammalian prey (compared to other owls) in the diets of *Strix uralensis* (Suzuki et al. 2013), *Strix hadorami* (Ben Dov, Atar, Baruchi, Levi, & Sapir, 2017), *Strix butleri* (Amr, Robb, Nunes, Abu Baker, & Walsh, 2016), *Strix occidentalis lucida* (Bravo‐Vinaja, Tarango‐Arambula, Clemente‐Sanchez, & Mendoze‐Martinez, 2005), *Strix rufipes* (Sergio Alavardo, Figueroa, Shehadeh, & Corales, 2007), and *Strix varia* (Wiens, Anthony, & Forsman, 2014), but not by the mammal‐dominated diet of *Strix occidentalis caurina* (Wiens et al., 2014). Nevertheless, even the in the case of *S. o. caurina*, the diet was studied in sympatry with a congener (*S. varia*), whose diet was taxonomically broad.

While our conclusion that whole genera of predators’ may specialize on prey taxa in contrast of the general predator–prey size trend relies on data from owls’ diets, it might apply in other guilds as well. Despite the long scientific interest in predator–prey size relations (e.g., Brose et al., 2006; Cohen et al., 1993; Hespenheide, 1973; Klompmaker et al., 2017; Naisbit et al., 2011; Nakazawa, 2017; Schoener et al., 1986), to the best our knowledge, this issue was not explicitly pointed out before. The relative ease of studying owl diets (i.e., by collecting pellets with mostly intact skeletal remains) allowed us not only to plan a geographically extensive study of multiple sympatric guild members, but also to corroborate our results using the rich owl diet literature. Given a similar research or a meta‐analysis focusing on different predator guilds, other deviations from the predator–prey size ratios trend may emerge. Paleontological studies of intraguild predator–prey size ratios could track the evolutionary histories and mechanisms of these specializations (e.g., when and in what environmental context did the lineage of modern‐day *Tyto* first started specializing on small mammals more than Strigid owls?).

In sum, prey selection by the owl species studied here, based on both predator size and taxonomic specialization, overrides variation due to species life history or environmental differences across space. While a general predator–prey size relationship has been previously demonstrated (e.g., Carbone et al., 1999; Costa, 2009; Hespenheide, 1973), it does not manifest in all ecosystems (e.g., Török, 1993). Disentangling the ecological or evolutionary causes of prey selection beyond body size thus warrants further research, which could shed more light on the fascinating subject of prey selection.

## CONFLICT OF INTEREST

None declared.

## AUTHOR CONTRIBUTIONS

OC and TD conceived the ideas and designed the methodology. OC collected and analyzed the data. OC and TD led the writing of the manuscript.

All authors contributed critically to the drafts and gave final approval for publication.
